# 超高效液相色谱-串联质谱法同时测定血浆与尿液中12种脂溶性贝类毒素

**DOI:** 10.3724/SP.J.1123.2020.11001

**Published:** 2021-04-08

**Authors:** Qiang LIN, Chao YANG, Meili LI, Jia WANG, Hanran HOU, Bing SHAO, Yumin NIU

**Affiliations:** 1.北京市延庆区疾病预防控制中心, 北京 102100; 1. Beijing Yanqing District Center for Disease Control and Prevention, Beijing 102100, China; 2.北京市疾病预防控制中心, 北京 100013; 2. Beijing Center for Disease Control and Prevention, Beijing 100013, China

**Keywords:** 超高效液相色谱-串联质谱, 脂溶性贝类毒素, 血浆, 尿液, ultra-high performance liquid chromatography-tandem mass spectrometry (UHPLC-MS/MS), lipophilic shellfish toxins, plasma, urine

## Abstract

生物样品中脂溶性贝类毒素的检测,可为食物中毒等突发公共卫生事件的流行病学调查以及中毒者的临床救治提供技术支持。目前的研究存在目标化合物少,以及方法前处理复杂、灵敏度低等问题。该研究通过优化前处理和色谱分离技术,建立了超高效液相色谱-串联质谱法测定血浆、尿液中12种脂溶性贝类毒素的方法。实验对提取试剂以及流动相的选择进行了优化,采用乙腈对尿液和血浆样品进行提取。采用Phenomenex Kinetex C18色谱柱(50 mm×3 mm, 2.6 μm)进行分离,以0.05%(v/v)氨水水溶液、90%(v/v)乙腈水溶液为流动相,以流速0.40 mL/min梯度洗脱时,12种目标化合物分离效果最好。串联质谱的离子源为电喷雾离子(ESI)源,采用多反应监测(MRM)模式检测。12种目标物的基质效应均在0.8~1.1之间,表明该前处理方法的基质干扰低,采用外标法可对化合物进行准确定量。12种贝类毒素的线性范围为0.03~36.25 μg/L,相关系数均大于0.995。尿液检测的方法定量限为0.23~0.63 μg/L,血浆检测的方法定量限为0.31~0.84 μg/L。3个加标水平的回收率为72.7%~124.1%,日内精密度为2.1%~20.0%,日间精密度为2.1%~15.3%。利用该方法检测健康人尿液和血浆样本,以及经腹腔注射12种贝类毒素的小鼠尿液和血液样本。20份健康人样本中未检出目标物,20份小鼠样本中12种贝类毒素均有检出。该方法操作简便,样品取样量少,方法灵敏高,适用于血浆和尿液中脂溶性贝类毒素的快速检测。

## Abstract (en)

Lipophilic shellfish toxins pose significant threats to the health of seafood consumers and public health. The symptoms of these kinds of toxins include severe diarrhea, abdominal cramps, nausea and gastrointestinal disorders. These symptoms could be hardly distinguished with many other symptoms of food poisoning and diseases. Therefore, a fast and accurate determination method in human biological samples is urgently needed for the accurate judgement of food poisoning incident, which is important for the investigation of public health emergencies and clinical treatment of poisoned patients. However, there were several flaws of the previous studies reported on the analysis of lipophilic shellfish toxins: (1) limited target compounds were covered; (2) the pre-treatment process was complex; (3) the sensitivity of the compound was low. In this study, a simple extraction method coupled with ultra-high performance liquid chromatography-tandem mass spectrometry (UPLC-MS/MS) was developed for the simultaneous determination of 12 lipophilic shellfish toxins, including azaspir acid 1 (AZA1), azaspir acid 2 (AZA2), azaspir acid 3 (AZA3), dinophysistoxin 1 (DTX1), dinophysistoxin 2 (DTX2), gymnodimine (GYM), hyessotoxin (HYTX), okadaic acid (OA), pinnatoxin (Pntx), pectenotoxins 2 (PTX2), spirolides 1 (SPX1), yessotoxin (YTX), in plasma and urine. Firstly, the instrument conditions were optimized. Different additions in mobile phase were compared and 0.05% (v/v) ammonia solution was selected since it can improve the peak shape of YTX and HYTX, and increase the respondence by four times. Secondly, the volume of acetonitrile (0.2, 0.4, 0.6, 0.8, 1.0 mL) use for the extraction of the target compounds in plasma was optimized. Satisfactory recoveries were obtained when 0.6 mL of acetonitrile was used. At the same time, satisfactory recoveries were obtained when 0.9 mL of acetonitrile was used in urine samples. Finally, under the optimized conditions, the 12 compounds in plasma and urine samples were ultrasonically extracted with acetonitrile. Chromatographic separation was performed on a Phenomenex Kinetex C18 column (50 mm×3 mm, 2.6 μm) with 90% (v/v) acetonitrile aqueous solution and water containing 0.05% (v/v) ammonia as mobile phases. Gradient elution with a flow rate of 0.40 mL/min was employed. The 12 compounds were monitored in the multiple reactions monitoring (MRM) mode with electrospray ionization (ESI) under both positive and negative conditions. The matrix effects of the 12 compounds ranged from 0.8 to 1.1. Therefore, external standard calibration curves were used for the quantification. The 12 shellfish toxins showed good linear relationship in the range of 0.03-36.25 μg/L with the correlation coefficients greater than 0.995. The limits of detection (LODs, *S/N*=3) were 0.08-0.21 ng/mL for the urine samples and 0.10-0.28 μg/L for the plasma samples, respectively. The limit of quantitations (LOQs, *S/N*=10) were 0.23-0.63 μg/L for the urine samples and 0.31-0.84 μg/L for the plasma samples, respectively. The recoveries of the 12 compounds were in the range of 72.7%-124.1% at three spiked levels (i. e., LOQ, three times LOQ, and ten times LOQ). The intra-day and inter-day precisions were 2.1%-20.0% and 2.1%-15.3%, respectively. The method was applied in the detection of the 12 lipophilic shellfish toxins in the urine and plasma samples of healthy humans and mice previously injected with the 12 shellfish toxins intraperitoneally. None of the 12 toxins were found in the samples from healthy human, while all of the 12 lipophilic shellfish toxins were found in the urine and plasma samples collected from the poisoned mice in the range of 1.14-2.35 μg/L and 1.01-1.17 μg/L, respectively. The established method has the advantages of sensitive, quick, easy to operate, and of low sample volume. It can be used for the simultaneous determination of 12 lipophilic shellfish toxins in urine and plasma samples.

贝类毒素亦称为藻毒素,由浮游植物自然产生,按照化合物性质主要分为脂溶性和水溶性两大类^[1]^。脂溶性贝类毒素主要包括大田软海绵酸毒素(OA)、鳍澡毒素(DTX)、扇贝类毒素(PTX)和原多甲藻毒素(AZAs)等毒素。相关研究表明,OA和DTX具有腹泻毒性,PTX具有肝毒性,误食脂溶性贝类毒素容易引起恶心、呕吐、腹泻等症状^[2,3,4,5]^。欧洲食品安全局(EFSA)对各类脂溶性贝类毒素及其亚型的食用安全计量、急性中毒剂量和限值都做了明确的规定^[6,7]^,如AZAs、PTX2总最大残留限量均为160.0 μg/kg,并根据新的毒理学数据将OA和DTXs的限量标准修订为45 μg/kg。目前我国没有相关限量标准。脂溶性贝类毒素中毒事件近几年在我国频繁出现,2010年9月~2019年5月,香港、澳门、珠海、宁波、福建、秦皇岛、唐山等地陆续出现70余起贝类毒素中毒事件^[8,9]^。

根据相关文献^[10,11,12,13,14]^报道,OA类毒素可长期蓄积于肠道等组织内,经组织吸收后主要以原型化合物的形式通过尿和粪便排出。针对生物样品中毒素的筛查有利于快速准确判断中毒原因。目前,我国关于脂溶性贝类毒素的检测标准有GB 5009.212-2016《食品安全国家标准贝类中腹泻性贝类毒素的测定》,该方法仅规定了贝类样品中OA、DTX1、DTX2等3种化合物的检测方法。针对生物样品中脂溶性贝类毒素的检测方法^[11,12,13,14]^较少,现有检测方法主要存在以下两方面问题:(1)生物样品中脂溶性贝类毒素以OA、DTX1、DTX2等3种化合物为主,目标化合物种类较少;(2)多采用固相萃取方法处理生物样品,前处理过程复杂,灵敏度低。为进一步完善突发公共卫生事件应急检测技术体系,亟须建立生物样品中脂溶性贝类毒素的检测方法,为食物中毒等突发公共卫生事件的流行病学调查以及中毒者的临床救治提供技术支持。

本方法以超高效液相色谱-三重四极杆质谱联用仪为研究平台,建立了简单、准确检测血浆、尿液中12种脂溶性贝类毒素的方法,可为食物中毒事件的处理提供技术支持。

## 1 实验部分

### 1.1 仪器与试剂

QTRAP 6500+超高效液相色谱-串联质谱仪(美国SCIEX公司);漩涡混合器(德国IKA公司);冷冻离心机(美国Eppdendorf公司); Milli-Q超纯水器(美国Millipore公司)。

乙腈、甲醇(LC-MS级,美国Sigma Aldrich公司);氨水(优级纯,美国Merck公司); 12种脂溶性贝类毒素标准物质均为溶解于甲醇的单标准溶液(见表1),购买于加拿大国家海洋研究中心,-18 ℃避光保存。

**表 1:** 12种脂溶性贝类毒素质谱参数及保留时间

Analyte	Abbreviation	Precursor ion (*m/z*)	Product ions (*m/z*)	Collision energies/eV	Declustering potential/V	Retention time/min	Ionization mode
Azaspir acid 1 (原多甲藻酸贝类毒素1)	AZA1	842.3	824.6^*^, 806.3	47, 60	90	3.24	+
Azaspir acid 2 (原多甲藻酸贝类毒素2)	AZA2	856.3	838.5^*^, 820.4	38, 55	42	3.38	+
Azaspir acid 3 (原多甲藻酸贝类毒素3)	AZA3	828.5	810.4^*^, 792.5	41, 53	100	2.73	+
Dinophysistoxin 1 (鳍藻毒素-1)	DTX1	817.3	255.1^*^, 113.1	-65, -95	-100	2.24	-
Dinophysistoxin 2 (鳍藻毒素-2)	DTX2	803.2	255.1^*^, 112.9	-63, -100	-100	1.96	-
Gymnodimine (环亚胺毒素)	GYM	508.2	490.4^*^, 392.2	34, 50	50	4.44	+
Hyessotoxin (类虾夷扇贝毒素)	HYTX	577.2	474.2^*^, 508.7	-42, -29	-60	2.01	-
Okadaic acid (大田软海绵酸)	OA	803.3	255.1^*^, 112.9	-61, -90	-200	1.82	-
Pinnatoxin (江瑶青毒素)	PNTX	694.5	164.2^*^, 458.4	62, 59	50	4.19	+
Pectenotoxins 2 (扇贝毒素)	PTX2	876.7	823.4^*^, 805.5	34, 37	100	4.69	+
Spirolides 1 (螺环内酯毒素)	SPX1	692.4	674.4^*^, 164.2	43, 59	60	4.85	+
Yessotoxin (虾夷扇贝毒素)	YTX	570.2	467.2^*^, 396.2	-41, -45	-135	2.00	-

* Quantitative ion.

### 1.2 溶液的配制

标准储备液:分别准确称取1 mL不同浓度标准品,用甲醇稀释20倍,配制成混合标准储备液,于4 ℃保存。标准使用液:将混合标准贮备液用初始流动相逐级稀释成标准使用液,浓度范围见表2。

**表 2:** 12种脂溶性贝类毒素的检出限、定量限、加标回收率和相对标准偏差(*n*=6)

Analyte	Linear range/ (μg/L)	LODs/(μg/L)			LOQs/(μg/L)			Average recoveries/%			Intra-day RSDs/%			Inter-day RSDs/%		
		Urine	Plasma		Urine	Plasma		Urine	Plasma		Urine	Plasma		Urine		Plasma
AZA1	0.03-16.25	0.09	0.12		0.27	0.36		74.8-102.2	76.1-101.2		6.4-12.0	8.2-15.0		6.4		10.0
AZA2	0.03-15.25	0.09	0.12		0.27	0.36		78.3-92.3	78.3-98.7		9.0-15.1	3.0-15.0		12.1		15.0
AZA3	0.03-13.00	0.08	0.10		0.23	0.31		78.4-101.2	78.5-98.7		2.1-15.1	2.5-12.0		9.1		8.2
DTX1	0.05-26.56	0.16	0.21		0.47	0.62		89.2-117.1	95.9-106.3		4.3-11.0	7.2-11.0		15.2		12.0
DTX2	0.05-23.75	0.15	0.20		0.45	0.60		74.6-88.3	72.7-97.1		3.3-15.6	3.4-15.0		11.3		15.0
GYM	0.06-31.25	0.18	0.24		0.54	0.72		97.4-119.3	91.4-117.7		8.7-16.0	4.5-18.0		9.3		3.0
HYTX	0.07-36.25	0.21	0.28		0.63	0.84		85.6-115.5	87.8-101.3		4.3-14.0	9.5-14.0		13.3		11.1
OA	0.05-26.25	0.15	0.20		0.45	0.60		88.2-120.0	85.3-115.3		7.9-18.0	13.0-14.0		15.3		12.2
PNTX	0.04-24.00	0.14	0.18		0.41	0.55		91.8-117.9	95.1-116.1		14.2-18.0	7.4-11.2		2.1		2.5
PTX2	0.05-27.50	0.15	0.20		0.45	0.60		91.5-124.1	99.4-123.8		7.7-17.4	9.4-20.0		4.3		8.7
SPX1	0.06-31.25	0.18	0.24		0.54	0.72		92.7-120.4	91.4-95.4		5.5-19.1	8.4-18.0		11.2		11.3
YTX	0.06-30.63	0.18	0.24		0.54	0.72		92.4-112.1	95.7-123.0		5.1-14.2	6.4-15.0		9.6		7.2

### 1.3 样品的采集

采用便利抽样招募捐赠者,所有受试者均了解此次研究的目的和意义,受试者签署同意书。小鼠经腹腔注射12种贝类毒素后收集尿液和血液。尿液样品使用棕色玻璃样品瓶收集。血液样品收集于含有肝素的5 mL采血管中,离心后取上层血浆。尿液和血浆放置于-20 ℃保存。

### 1.4 样品前处理

1.4.1 尿液样品的制备

取0.3 mL尿液,加入0.9 mL乙腈后涡旋1 min,以9000 r/min离心10 min,吸取0.4 mL上清液,加入0.4 mL超纯水,涡旋30 s,过0.22 μm有机滤膜后上机检测。

1.4.2 血浆样品制备

取0.2 mL血浆,加入0.6 mL乙腈后涡旋1 min,以9000 r/min离心10 min,吸取0.4 mL上清液,加入0.4 mL超纯水,涡旋30 s,过0.22 μm有机滤膜后上机检测。

### 1.5 仪器条件

1.5.1 色谱条件

色谱柱:Phenomenex Kinetex C18(50 mm×3 mm, 2.6 μm);流动相:0.05%(v/v)氨水水溶液(A相)-含0.05%(v/v)氨水的90%(v/v)乙腈水溶液(B相)。梯度淋洗程序:0~0.50 min, 70%A; 0.50~4.00 min, 10%A; 4.00~5.50 min, 10%A; 5.50~5.60 min, 70%A; 5.60~6.10 min, 70%A。流速:0.40 mL/min;柱温:35 ℃;进样量:5 μL。

1.5.2 质谱条件

电喷雾离子源;正负离子扫描模式;多反应监测(MRM)模式采集。喷雾电压:±4500 V,离子源温度:550 ℃,碰撞气压力:Medium;气帘气压力:0.21 MPa(30 psi);雾化气压力GS1和辅助加热器压力GS2:0.38 MPa (55 psi)。其他质谱参数见表1。MRM图谱见图1。

**图 1:**
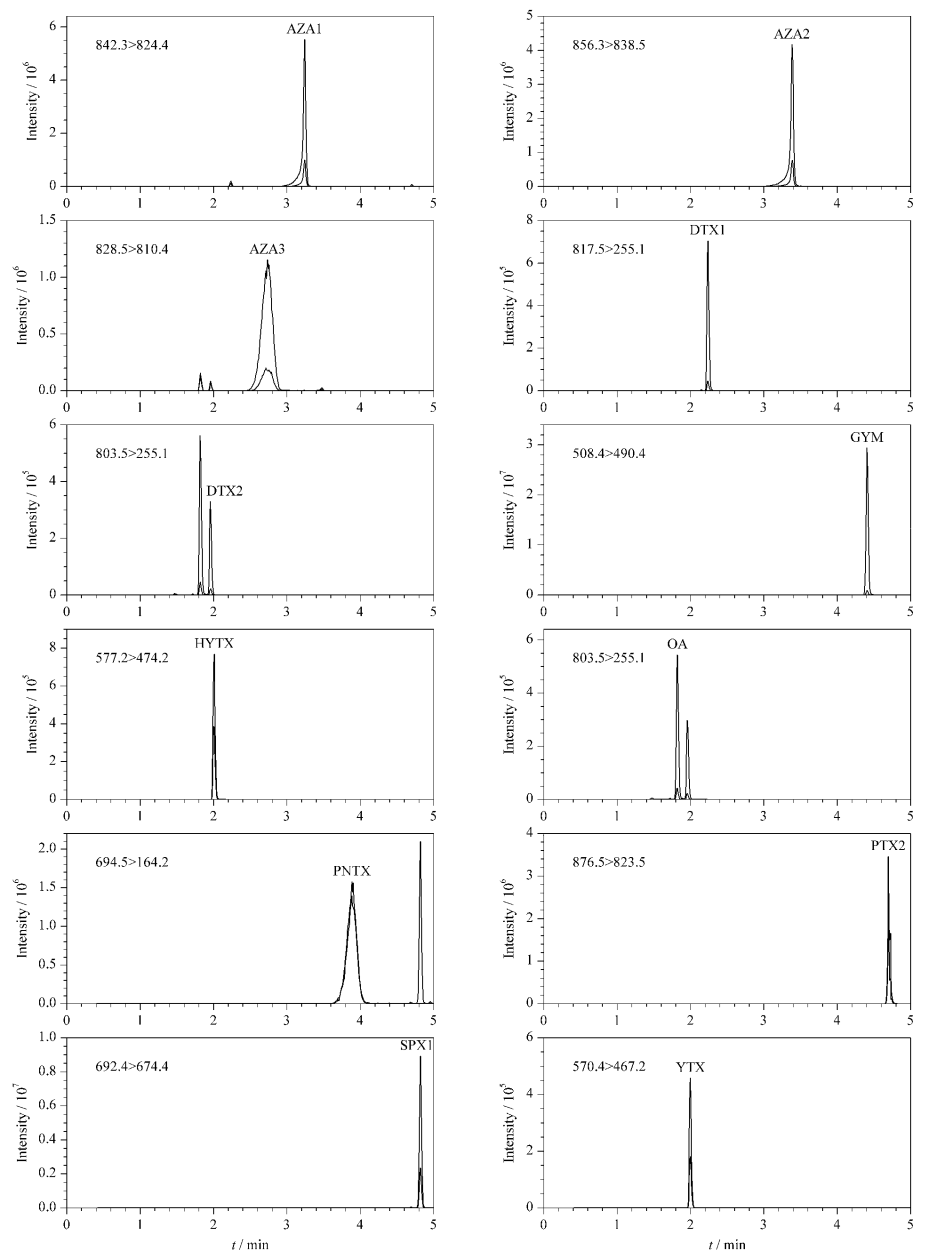
血浆加标(1.0 μg/L)样品中12种脂溶性贝类毒素的MRM谱图

## 2 结果与讨论

### 2.1 UPLC-MS/MS条件的优化

目前检测脂溶性贝类毒素的流动相体系主要有酸性体系和碱性体系两种^[2]^。在酸性条件下(流动相A为0.1%(v/v)甲酸+2 mmoL/L甲酸铵溶液,流动相B为0.1%(v/v)甲酸乙腈溶液), YTX、HYTX的峰形出现拖尾,响应较低(见图2a)。在碱性条件下(流动相A为0.1%(v/v)氨水,流动相B为90%(v/v)乙腈+0.1%(v/v)氨水), ESI^-^灵敏度提高,YTX、HYTX拖尾现象明显改善,响应值提高4倍(见图2b)。

**图 2:**
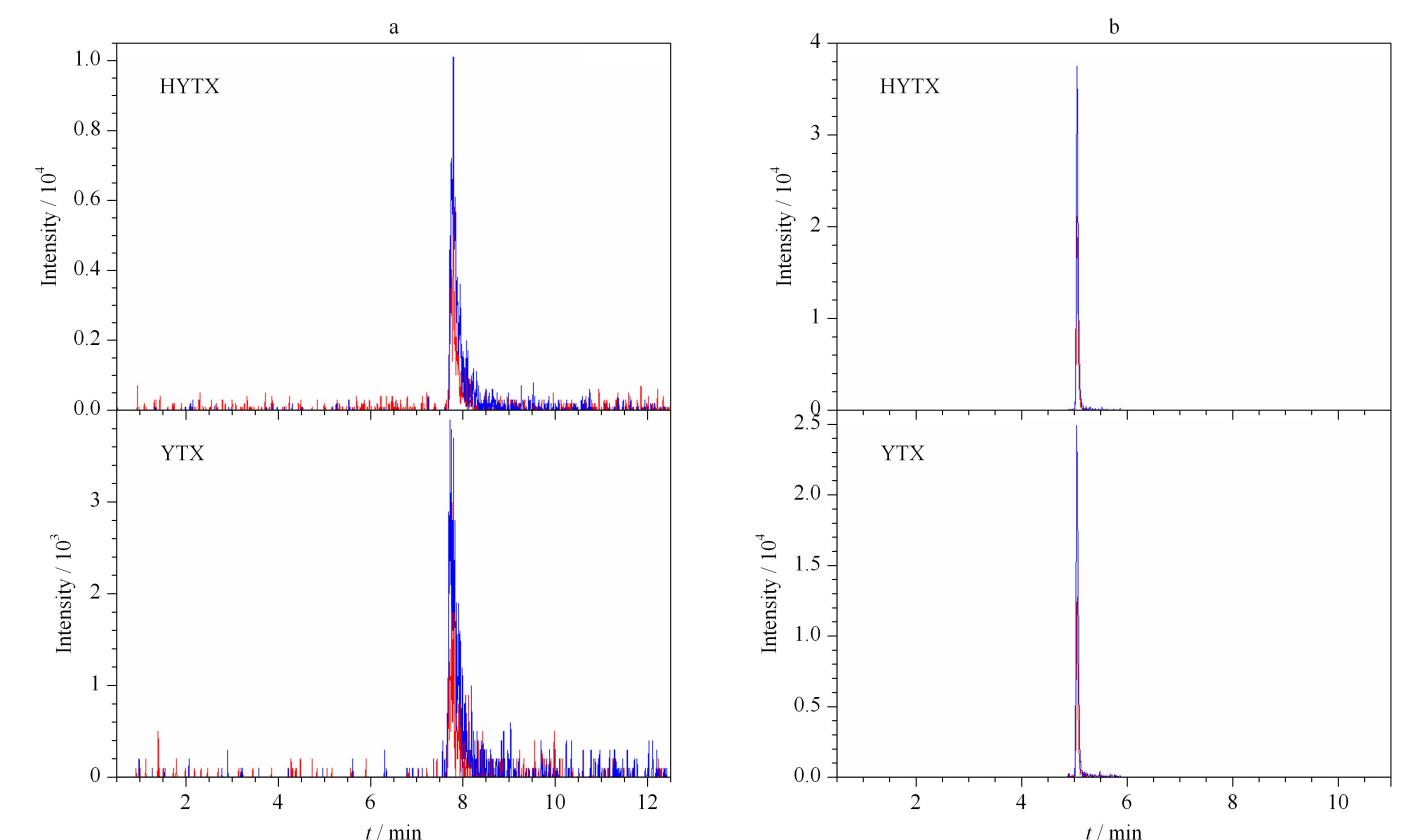
不同流动相体系中YTX(0.23 μg/L)和HYTX(0.30 μg/L)的总离子流图

进一步比较了流动相中氨水的体积分数(0.02%、0.05%、0.1%)对化合物响应值的影响。当流动相中含0.05%(v/v)氨水时,除PTX2外,其余化合物的响应值最大。最终,选择流动相A为0.05%(v/v)氨水,流动相B为含0.05%(v/v)氨水的90%(v/v)乙腈水溶液。

### 2.2 样品前处理条件的优化

对于生物样本,乙腈是最常用的提取溶剂。本研究考察了不同体积(0.2、0.4、0.6、0.8、1.0 mL)的乙腈对血浆样品中12种贝类毒素的提取效果,结果如图3所示。0.2 mL和0.4 mL的乙腈提取效率较低,为30%~69%;当乙腈提取液的体积升高至0.6 mL时,提取效率为78%~121%;乙腈体积继续升高,提取效率没有进一步改善。因此,最终确定乙腈提取液的体积为0.6 mL。

**图 3:**
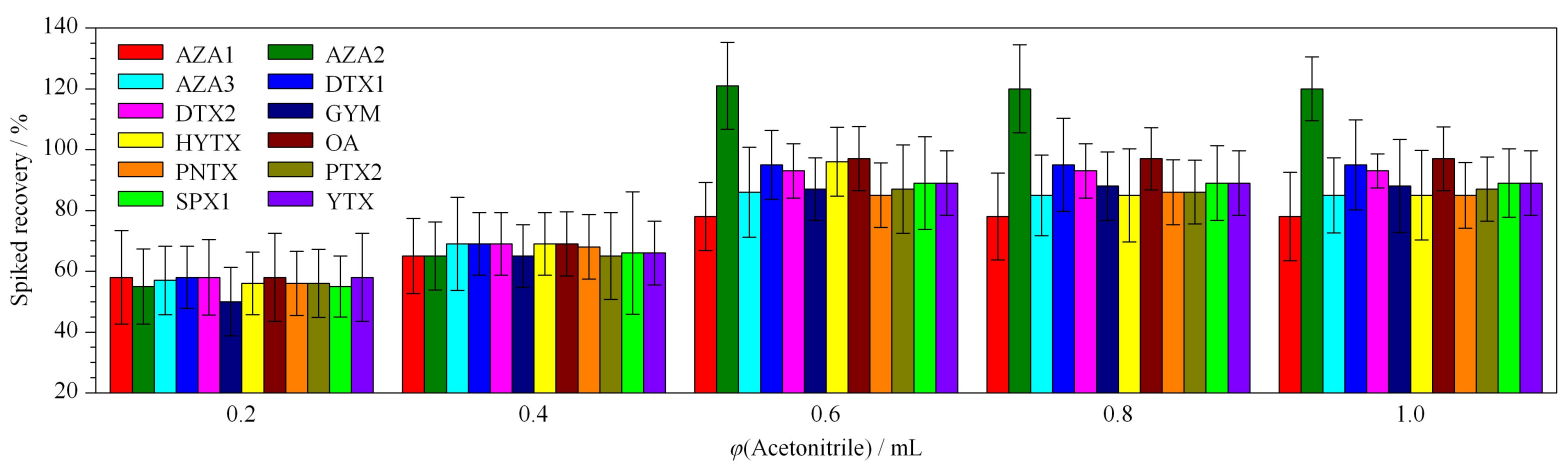
不同体积的提取试剂对血浆中12种脂溶性贝类毒素加标回收率的影响(*n*=4)

### 2.3 方法学验证

2.3.1 基质效应

基质中存在的干扰成分会产生基质增强或基质抑制效应,进而影响目标化合物的定量^[15,16,17,18]^。为评估基质效应,分别采用空白尿液基质提取液、空白血浆基质提取液配制12种化合物的基质校准曲线,两种曲线方程的斜率与溶剂标准曲线方程的比值均在0.8~1.1之间,表明该前处理方法的基质干扰低,采用溶剂标准曲线可对化合物进行准确定量。

2.3.2 标准工作曲线和灵敏度

将混合标准溶液按照上述色谱、质谱条件进行检测,结果表明,12种化合物在0.03~36.25 μg/L范围内线性关系良好,相关系数(*r*^2^)均大于0.995。

以检出限(LOD)和定量限(LOQ)评估方法的灵敏度。在尿液和血浆中添加目标化合物,信噪比(*S/N*)为3:1时确定方法的LOD为0.09~0.28 μg/L,当*S/N*为10:1时确定方法的LOQ为0.23~0.84 μg/L(见表2)。

2.3.3 方法的精密度和准确度

空白尿液和空白血浆中分别加入LOQ、2倍LOQ、10倍LOQ等3个水平的目标化合物,每个水平进行6次平行试验,加标回收率的相对标准偏差(RSD)作为日内精密度。2倍LOQ的加标水平进行5 d日间精密度测定。方法的回收率为72.7%~124.1%,日内精密度为2.1%~20.0%,日间精密度为2.1%~15.3%(见表2)。

### 2.4 样品的测定

利用本方法检测了10份健康人尿液样本和10份健康人血浆样本,以及经腹腔注射12种脂溶性贝类毒素的小鼠尿液和血浆样品。10份人尿液样本和10份血浆样本均未检出12种脂溶性贝类毒素。在10份小鼠尿液样本中,均检测出12种贝类毒素,含量在1.14~2.35 μg/L之间;在10份小鼠血浆样品中,同样检测出12种贝类毒素,含量在1.01~1.17 μg/L之间。

## 3 结论

本研究分别建立了尿液和血浆中12中脂溶性贝类毒素的检测方法,取样量少,操作简单快速,灵敏度高,适合尿液和血浆中脂溶性贝类毒素的检测。
